# Environmental Regulation Intensity, Carbon Footprint and Green Total Factor Productivity of Manufacturing Industries

**DOI:** 10.3390/ijerph19010553

**Published:** 2022-01-04

**Authors:** Lei Wang, Yu Yan

**Affiliations:** Department of Regional Economics, Central China Development Institute, Wuhan University, Wuhan 430072, China; 2019206320001@whu.edu.cn

**Keywords:** SBM-MALMQUIST model, environmental regulations, direct carbon footprint

## Abstract

In terms of the development of the manufacturing industry, the Chinese government has carried out environmental regulations and set up production standards for related industries. This is an environmentally-friendly and economic action, which is also in line with the requirements of building a green economy for China. Meanwhile, whether from the micro regulatory measures or the macro government policies, carbon emission is an inevitable problem in the study of environmental problems. This paper will explore the impact of environmental regulation on the green economy based on carbon emissions and study the optimal environment regulation intensity that relates to a direct carbon footprint under the maximum green economic benefits. A SBM-MALMQUIST model is established to measure the green total factor productivity according to 27 Chinese manufacturing industries through the MAXDEA software. It is found that the intensity of environmental regulation has a significant impact on green total factor productivity, and direct carbon footprint also exhibits a partial intermediary effect, participating in the mechanism that affects green total factor productivity. Combined with the industrial characteristics and the above research results, this paper puts forward the adjustment strategy of reasonable environmental regulation for the manufacturing industry, which conforms to the national policy guidance, and will be beneficial in promoting the economic development of the green manufacturing industry.

## 1. Introduction

Since China’s reform and opening up, the manufacturing industry has maintained a trend of rapid development. In 2019, China’s industrial added value reached RMB 31.7 billion, among which the added value of manufacturing topped the world in both volume and growth. However, the rapid growth of the manufacturing industry brings about excessive waste of resources and serious loss of environmental benefits, which brings constant pressure on the environmental protection. The average annual energy consumption of the industrial sector is more than 80% of the total national energy consumption and greenhouse gas emissions are the main form. In the recent three global environmental performance index (EPI) rankings, China ranked 176th, 120th and 116th, respectively. It not only reflects the current serious environmental problem in China, but also illustrates the weak environmental regulation in China’s manufacturing industry. At the same time, the emphasis upon environment has been on the rise. The 14th Five-Year Plan clearly states that China will promote high-quality development with high-quality ecological environmental protection, adhere to ecological priority and green development, give full play to the role of ecological and environmental protection in optimizing and adjusting economic and social development, and promote the deepening transformation of economic and social development. How to achieve a win-win situation of environmental protection and economic development has always been a hot issue in recent years. For the manufacturing industry, the balance between the two lies in the intensity of environmental regulation. According to the literature, the current research mainly holds two views. Some scholars believe that environmental regulation greatly limits technical efficiency, and the negative impact on economic benefits is greater than the promotion of environmental benefits [1], which means the reduction of market share and the weakening of market competitiveness caused by the expansion of production costs for the company [2]. Some others believe that the intensity of environmental regulation within a reasonable range can promote the improvement of economic and environmental benefits at the same time. China’s industries have always followed the principle of the priority of the economy. Some literature shows that environmental regulations implemented by the government, such as the environmental tax policy, can improve the efficiency of technological progress of enterprises [3], or verifies that environmental regulation promotes the overall innovation (i.e., the integration of production technology innovation and pollution control technology innovation) [4,5,6,7]. However, with the increasing efforts of domestic environmental control and the growing economic penalties for environmental pollution, we have to investigate the changes of green total factor productivity (the evaluation system of green total factor productivity not only considers the traditional capital and labor factors, but also considers the resource and environmental factors that have a great impact on economic development, which can more accurately measure the contribution of total factor productivity to green economic growth, hereinafter called the ‘GTFP’) [8], its impact on economic development can be quantitatively analyzed so as to obtain the intensity of environmental regulation for the optimal economic development with consideration of economic development and environmental protection. Many scholars used different methods to measure the intensity of environmental regulation to verify the impact of the economic benefits of enterprises (mainly in productivity and pollution reduction cost) and found a positive correlation [9,10,11,12]. At present, under the guidance of China’s mainstream policy of environmental protection, the manufacturing industry bears the brunt of exploring the optimal GTFP of the industry.

The key is to how to design a measurement of environmental regulation intensity. For existing research, the measurement of environmental regulation intensity is not reasonable enough. There are natural differences in production structure and industrial nature among industries, so the general calculation and analysis can be too rough; many scholars only take manufacturing pollution waste (e.g., waste water, waste gas, solid waste) as the main index to classify industries, ignoring the environmental impact of manufacturing resource consumption, which is not comprehensive, either. All of the above results lead to a large deviation between the classification results and the actual environmental damage of various industries. On the other hand, there is a lack of in-depth analysis of the core mechanism on the impact, that is, whether reasonable environmental regulation leads to the improvement of GTFP under the regulation. If yes, how? The relevant research and analyses are too simple to clarify the impact of the mechanism of industry, the market and environmental regulation factors on the GTFP. They ignore the causal inference of environmental regulation effect and fail to provide more accurate quantitative analysis for the evaluation of the environmental regulation effect.

At the same time, some scholars point out that reasonable government intervention or regulation will greatly promote the improvement of the environment [13,14,15]. Some believes that productivity and pollution rate of an industry show a positive correlation to the cost of environmental regulation [16,17,18,19]. Marconi noted that some environmental protection policies can speed up technological improvement and environmental pollution reduction [20]. Although many scholars suggest through examples that government should take the lead in the environmental regulation of manufacturing industry, specific and effective suggestions are lacking [21,22,23,24,25,26,27]. Therefore, this paper uses the panel data of 27 manufacturing industries in China from 2009 to 2016 to verify the impact of environmental regulation intensity on GTFP by means of linear regression to study how a win-win situation can be achieved. Meanwhile, against the background that achieving “carbon peak” and “carbon neutrality” has been taken as one of the key tasks in 2021 since China’s central economic work conference in 2020, controlling the absolute value of carbon emissions has become urgent. Traditionally, while the manufacturing sector drives economic growth [28,29,30,31,32], it is often accompanied by increased carbon emissions and large amounts of greenhouse gases and pollutants [33,34,35,36]. This paper integrates carbon emissions into the mechanism of environmental regulation intensity affecting GTFP, and further explores whether the control of carbon emissions can improve the comprehensive environmental and economic performance of the manufacturing industry. The paper mainly finds that the scale of environmental regulation is different in the three types of industries divided by the degree of pollution. For light pollution industries, the intensity of environmental regulation should be improved to enhance GTFP; for moderate polluting industries, whether to improve or reduce the intensity of environmental regulation depends on the actual situation; for heavy pollution industries, the intensity of environmental regulation should be reduced in order to improve the comprehensive benefits of the environment and economy. Firstly, based on the degree of pollution, the paper deeply studies the reasons for GTFP differences in manufacturing industries across the country, and carries out analysis with industry differences to endow the practice of environmental regulation with more practical significance. In accordance with the current policy, this paper then creatively adds the carbon footprint as an intermediary variable to the research of China’s manufacturing environmental regulation and GTFP, which is conducive to the manufacturing industry to achieve more smooth environmental regulation with the help of the government.

The paper consists of five sections. Section 1 is the literature review of the impact of environmental regulations on enterprises. The method of calculating GTFP calculation based on MAXDEA is described in Section 2. In Section 2, the method to calculate the environmental regulation intensity is discussed. Empirical results are given in Section 4. Conclusions and policy recommendations are discussed in Section 5.

## 2. GTFP Calculation Based on MAXDEA

**Figure 1:**
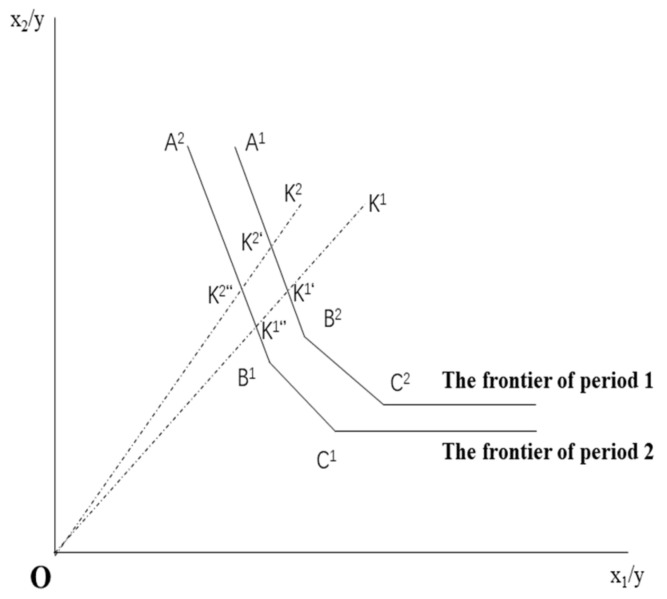
Diagram of Malmquist productivity index.

The GTFP of the manufacturing industry is measured through data on input and output. The data are from the National Bureau of Statistics, *China Industrial Statistical Yearbook* and *China Energy Statistical Yearbook*. The main data and nouns in this paper are explained in Table 1 below:

Through MAXDEA software (The manufacturer of the software is Beijing Rewomadi Software Co., LTD., from Beijing, China), the paper employs the cross-referencing of adjacent fronts (geometric average of two malmquists). To analyze the technical efficiency change of the evaluated index, we need to refer to the production frontier to get its efficiency in two periods (the index selected in this paper is from 2009 to 2016, so the measurement efficiency of 2010 to 2016 can be obtained). For the production frontier of period 1 and 2 please see Figure 1.

Frontier 1: Malmquist technology rate index is:

(1) $$E^{1}\left(K^{1}\right)=\frac{OK^{1^{\prime }}}{OK^{1}}$$

(2) $$E^{1}\left(K^{2}\right)=\frac{OK^{2^{\prime }}}{OK^{2}}$$

(3) $$\frac{E^{1}\left(K^{1}\right)}{E^{1}\left(K^{2}\right)}=\frac{OK^{2^{\prime }}/OK^{2}}{OK^{1^{\prime }}/OK^{1}}$$

Referring to Frontier 2, Malmquist’s productivity index is:

(4) $$E^{2}\left(K^{1}\right)=\frac{OK^{1^{\prime \prime }}}{OK^{1}}$$

(5) $$E^{2}\left(K^{2}\right)=\frac{OK^{2^{\prime \prime }}}{OK^{2}}$$

(6) $$\frac{E^{2}\left(K^{1}\right)}{E^{2}\left(K^{2}\right)}=\frac{OK^{2^{\prime \prime }}/OK^{2}}{OK^{1^{\prime \prime }}/OK^{1}}$$

The E reference set (evaluated index) represents the efficiency value of the DEA model. The superscript “1” represents the evaluation index of the reference set as period 1, and the superscript of “2” indicates the evaluation index of the reference set as period 2. The projection of the evaluated index on frontier 1 is indicated by ′ and the projection on frontier 2 is indicated by ″.

Referring to frontier 1 and frontier 2, two Malmquist indexes are obtained, adopted the geometric mean of the two Malmquist indexes as the Malmquist index of the evaluated index, i.e.,

(7) $$M_{ac}K^{2},\;K^{1}=\sqrt{\frac{E^{1}\left(K^{2}\right)E^{2}\left(K^{2}\right)}{E^{1}\left(K^{1}\right)E^{2}\left(K^{1}\right)}}=\sqrt{\frac{OK^{2^{\prime }}OK^{2^{\prime \prime }}/OK^{2}OK^{2}}{OK^{1^{\prime }}OK^{1^{\prime \prime }}/OK^{1}OK^{1}}}$$

The Malmquist index from period *t* to *t* + 1 is represented as:

(8) $$M_{ac}x^{t+1},\;y^{t+1},x^{t},y^{t}=\sqrt{\frac{E^{t}\left(x^{t+1},\;y^{t+1}\right)\;E^{t+1}\left(x^{t+1},\;y^{t+1}\right)}{E^{t}\left(x^{t},y^{t}\right)\;E^{t+1}\left(x^{t},y^{t}\right)}}$$

In Malmquist’s formula, *E^t^* (*x^t^*, *y^t^*) and *E^t^*^+1^ (*x^t^*^+1^, *y^t^*^+1^) are the technical efficiency values of K in two periods respectively. We regarded them as the technical efficiency changes in two periods:

(9) $$EC=\frac{E^{t+1}\left(x^{t+1},\;y^{t+1}\right)}{E^{t}\left(x^{t},y^{t}\right)}$$

The movement of frontier 2 compared to frontier 1 may be made from:

(10) $$OK_{1}^{\prime }/OK_{1}^{\prime \prime }=E^{1}\left(x^{1},y^{1}\right)/E^{2}\left(x^{1},y^{1}\right)$$

and also

(11) $$OK_{2}^{\prime }/OK_{2}^{\prime \prime }=E^{1}\left(x^{2},y^{2}\right)/E^{2}\left(x^{2},y^{2}\right)$$

A ratio greater than 1 indicates the frontier moves forward, and less than 1 indicates the frontier moves backward. The forward movement of frontier represents technological progress. We can use geometric mean calculation as a technical change:

(12) $$TC_{ac}=\sqrt{E^{t}\left(x^{t},y^{t}\right)E^{t}\left(x^{t+1},y^{t+1}\right)/\left[E^{t+1}\left(x^{t},y^{t}\right)E^{t+1}\left(x^{t+1},y^{t+1}\right)\right]}$$

The quantitative relationship among Malmquist index, efficiency change and technology change is expressed as *MI* = *EC* ∗ *TC*. The Malmquist index, or the green total factor rate, can be

(13) $$\begin{array}{l} M_{ac}=\sqrt{\frac{E^{t}\left(x^{t+1},\;y^{t+1}\right)\;E^{t+1}\left(x^{t+1},\;y^{t+1}\right)}{E^{t}\left(x^{t},y^{t}\right)\;E^{t+1}\left(x^{t},y^{t}\right)}}\\ =\frac{E^{t+1}\left(x^{t+1},\;y^{t+1}\right)}{E^{t}\left(x^{t},y^{t}\right)}\sqrt{E^{t}\left(x^{t},y^{t}\right)\;E^{t}\left(x^{t+1},y^{t+1}\right)/\left[E^{t+1}\left(x^{t},y^{t}\right)\;E^{t+1}\left(x^{t+1},y^{t+1}\right)\right]} \end{array}$$

The calculated GTFP of various industries is shown in the Table 2 below.

## 3. Calculation of Environmental Regulation Intensity

In order to more reasonably analyze the level of environmental regulation intensity of manufacturing industries, referring to Li Ling’s method, we average the value of wastewater, waste and exhaust gas of each industry unit output value to get the calculated environmental loss intensity of manufacturing industries, and we divide them into three categories based on the size of values: light pollution industry, moderate pollution industry and heavy pollution industry.

The lack of unified government environmental intervention system or clear independent regulatory tools lead to the difficulty of measurement for China. With the improvement of environmental protection efforts, the emphasis of policies on environmental benefits and the GTFP proposed by scholars in recent years, it is generally believed that the investigation of an industry should include both environmental and economic benefits. We often say that “Lucid waters and lush mountains are invaluable assets”. For the industry, under reasonable environmental regulations, it will improve the development efficiency of the green economy. On the one hand, due to the significant increase of pollution cost, the regulation will reduce the pollution emission intensity of enterprises. On the other hand, enterprises can obtain the same economic benefits with lower unit environmental cost through the improvement of technical efficiency, which is also a form of green. This paper considers the impact of environmental regulation on GTFP to obtain the optimal intensity of environmental regulation among industries.

The scientific selection of environmental regulation intensity is based on the objective reflection of various industries. From the literature at home and abroad, there are three main measurement methods:

1. To measure the intensity of environmental regulation by the number of industrial environmental policies. The disadvantage is that the environmental constraints are different between different industries and policies, which cannot be systematically measured, making the method too rough.
2. To measure the pollution discharge of each industry. The disadvantage is that it is easy to be affected by the scale of the industry, nor can it reflect the environmental standards set by enterprises in the pollutant discharge treatment. The policy sets different environmental standards for different industries, which affect the cost of pollutant discharge.
3. To use the per capita income of each industry as a measure of the intensity of environmental regulation. This view holds that the higher the per capita income in an industry, the higher the requirements for environmental regulation. However, environmental regulation affects the environmental cost of industries with no significant relationship with economic benefits. For high-tech industries with high incomes and low environmental pollution, such a view is one-sided and unconvincing.

This article examines 27 manufacturing industries. Due to the differences in the nature of industries and the different degrees of pollution of various emissions to the environment, the previous research is not comprehensive enough. Referring to the existing research [12], this paper uses the hierarchical analysis method to establish the index level, target level and basic level to sort out the environmental regulation intensity among various industries, as is shown in Table 3 below:

The intensity of environmental regulation can be measured by three single indicators, i.e., the standard rate of wastewater discharge, the removal rate of sulfur dioxide, and the comprehensive utilization rate of solid waste.

The standard rate of wastewater discharge, the removal rate of sulfur dioxide and the comprehensive utilization rate of solid waste of manufacturing industries from 2009 to 2015 in the *China Industrial Statistical Yearbook* are normalized to eliminate the immeasurability of the data.

Due to the differences in the degree of environmental pollution caused by wastewater, solid waste and waste gas, there are different levels of regulation to indicators used to measure the intensity of environmental regulation among industries. The addition of equal weights can cause greater errors in the regulation intensity and the actual situation. In this paper, different weights are given to each index, and the adjustment coefficient W_*j*_ is calculated with reference to [12].

$$W_{ij}={\mathrm{UI}}_{ij}/{\bar{\mathrm{UI}}}_{j}$$

${\bar{\mathrm{UI}}}_{j}$ indicates the national average of pollutant J’s emission per unit output value.

**Figure 2:**
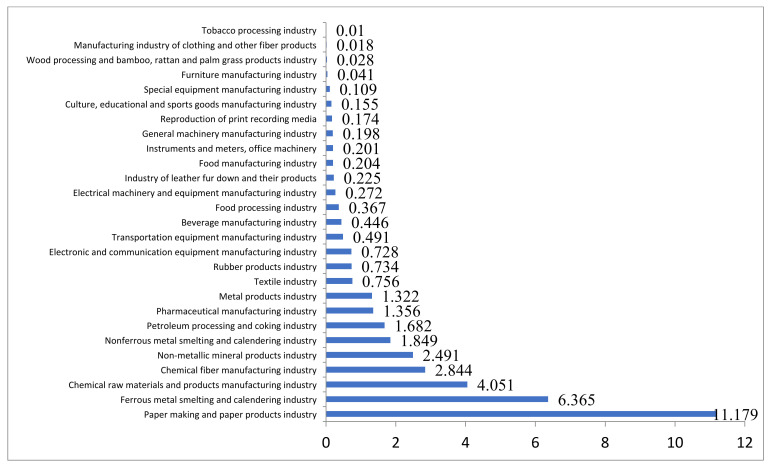
Environmental regulation intensity of various industries.

**Figure 3:**
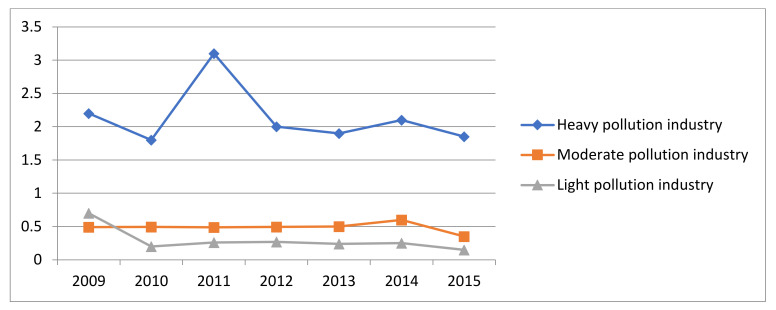
Average regulation intensity of the three major categories of pollution industries.

According to the standardized value and average weight of each index, the environmental regulation intensity of each individual index is calculated, and the total regulation intensity of each industry is obtained by summing up all kinds of single indexes for each industry. Detailed data are shown in Table 4 below:

From the Figure 2 and Figure 3, we can draw the following conclusions. First, as traditional heavy pollution industries, the paper making and paper products industry, the ferrous metal processing industry and the chemical raw material manufacturing industry are under greater environmental regulation, bearing the first-tier environmental regulation intensity. Second, in addition to the electronic manufacturing industry, food processing industry and other light polluting industries in the medium intensity of regulation, moderate pollution industries and light pollution industries are generally in the second and third echelon of the intensity of environmental regulation, respectively. In general, the higher the pollution level of the industry, the higher the intensity of environmental regulation. The average regulation intensity of heavy pollution industries (2.14) was significantly higher than that of moderate pollution industries (0.480) and light pollution industries (0.200). The overall intensity trend of the three pollution industries remained stable from 2009 to 2015.

## 4. Empirical Results

GTFP reflects the comprehensive measurement of economic and environmental benefits of the industry. At the same time, this paper takes the carbon footprint (To simplify the research, this paper takes the direct carbon footprint of manufacturing industry as the main measurement object. Data source: *China Energy Statistical Yearbook*. The calculation process and results are shown in the appendix) of various industries as an intermediary variable to explore the deep mechanism of environmental regulation affecting GTFP, that is, whether to improve the environmental benefits through the reduction of carbon emissions on the carbon footprint, and thus improve the GTFP. Beyond that, considering the differences among industries, the following three control variables are selected in this paper to obtain robust estimates: the ratio of cost-profit (RCP), full-staff labor productivity (LP) and energy productivity (EP). (1) RCP: This reflects the ratio of cost input and profit in various industries. (2) LP: The ratio of industrial added value to all employees in the corresponding industry reflects the average value created in each industry per capita every year; (3) EP: The ratio of industrial added value to the energy consumption of the corresponding industry. The data are all from the China Industrial Statistical Yearbook, and the missing data in some years are supplemented by the interpolation method. The above three groups of control variables can well reflect the economic or environmental conditions among industries, thus obtaining more accurate estimates. The following six sets of benchmark regression models were established (The subscript “i” indicates the i-th industry, and subscript “t” indicates the t-th year).

(14) CF_it_ = C_0_ + C_1_ER_it_ + ε_it_

(15) GTFP_it_ = C_0_ + C_1_ER_it_ + ε_it_

(16) GTFP_it_ = C_0_ + C_1_ER_it_ + C_2_CF_it_ + ε_it_

(17) GTFP_it_ = C_0_ + C_1_ER_it_ + C2CF_it_ + C_3_RCP + ε_it_

(18) GTFP_it_ = C_0_ + C_1_ERit + C_2_CFit + C_3_RCP + C_4_LP + ε_it_

(19) GTFP_it_ = C_0_ + C_1_ER_it_ + C_2_CF_it_ + C_3_RCP + C_4_LP + C_5_EP + ε_it_

Through the results of benchmark regression (Table 5), we can find that the environmental regulation in the manufacturing industry has a significant impact on its GTFP. In the first and third models, the regression coefficient of the carbon footprint is positive and significant at the significant level of 1%, which indicates that the direct carbon footprint of the industry has a partial mediating effect therein.

In order to discuss the optimal intensity of environmental regulation in different industries in detail, this paper divides the industries into three categories (light, moderate and heavy pollution industries) for regression analysis. Descriptive statistics are shown in Table 6 below:

Three sets of panel data models are set up to examine the correlation among environmental regulation intensity, GTFP and carbon footprint of various industries.

(20) CF_it_ = C_0_ + C_1_ER_it_ + ε_it_

(21) TFP = C_0_ + C_1_ER_it_ + C_2_ER^2^_it_ + C_3_RCP + C_4_LP + C_5_EP + ε_it_

(22) TFP = C_0_ + C_1_ER_it_ + C_2_ER^2^_it_ + C_3_RCP + C_4_LP + C_5_EP + C_6_CF_it_ + ε_it_

The regression results are shown in the following table.

Comparing the regression results by classification and the benchmark regression, it can be found that in regression (2), the coefficient of environmental regulation intensity of light and moderate pollution industries is significantly increased, while that of heavy pollution industries decreased slightly. It can be inferred that in the whole manufacturing industry, the intensity of environmental regulation has a greater impact on GTFP in light and moderate pollution industries. Through formula (1), we can find that there is a significant correlation between environmental regulation intensity and direct carbon footprint in light and moderate pollution industries, and the coefficient has a significant increase compared with the benchmark regression, indicating that the impact of direct carbon footprint on environmental regulation intensity is mainly concentrated in light and moderate pollution industries. At the same time, we can see that the regression coefficient between environmental regulation intensity and the direct carbon footprint of heavy pollution industries is not significant, indicating that there is no mediating effect.

By further analyzing the econometric regression results in Table 7, we can find that, without considering carbon footprint as an intermediary variable, the regression estimation results of environmental regulation intensity and GTFP show that the primary and secondary coefficients of environmental regulation intensity of the three major industries are positive and negative respectively, and are statistically significant. This shows that the intensity of environmental regulation and GTFP present an inverted “U” shape. For heavy pollution industries, the inflection point is that the intensity of environmental regulation reaches 0.426. The environmental regulation intensity of heavy pollution industries is greater than 1, and the range does not include the inflection point. It shows that the strong current environmental regulation intensity, aggravating the burden of enterprises and exceeding the bearing capacity of enterprises. Although the environmental benefits of the industry have been improved, the adverse impact on the economic development of such industries is dominant, resulting in the decrease of GTFP with the improvement of environmental regulation intensity. According to the results of econometric analysis, the environmental regulation of the industry should be appropriately relaxed to reduce the regulation intensity. For the light pollution industry, the inflection point is that the intensity of environmental regulation is equal to 1.381, and the intensity of environmental regulation in the light pollution industry is less than 0.5, the range of which excludes the inflection point. At present, the relevant departments have weak control on and insufficient attention to the light pollution industries, with high-tech industries and clean industries as the main body, resulting in the low intensity of environmental regulation at the current stage. Attention needs to be increased and the intensity of relevant environmental regulations strengthened. Appropriate improvement of the environmental regulation intensity in such industries will help to improve the GTFP. For moderate pollution industries, the inflection point is that the environmental regulation intensity is equal to 0.837. The regulation intensity range of the moderate pollution industry includes this inflection point, indicating that the environmental regulation intensity of such industries can be adjusted to achieve the optimal environmental regulation intensity.

Based on the in-depth study of the mechanism of the effect of environmental regulation intensity on GTFP, we take carbon footprint as an intermediary variable for econometric analysis. It can be found that the impact of environmental regulation intensity on the carbon footprint is significant in the light and moderate pollution industries, with coefficients of 0.764 and 2.579, which are significant at the significance level of 10% and 1%, respectively. Further analysis shows that, after taking the carbon footprint as an independent variable, the impact of the carbon footprint and environmental regulation intensity on green TFP is significant in the light pollution industry, and the coefficient of environmental regulation intensity has changed significantly. It shows that the carbon footprint plays a partial intermediary effect on the light pollution industry. In moderate pollution industries, the impact of the carbon footprint on GTFP is significant, while the environmental regulation intensity becomes insignificant, which indicates that carbon footprint also plays a partial intermediary effect on moderate pollution industries. Therefore, moderate and light pollution industries can control the intensity of environmental regulation by increasing or reducing the carbon emission of energy in the industry. For heavy pollution industries, the mediating effect of the carbon footprint is not significant.

## 5. Research Conclusions and Policy Recommendations

The current policy background of China is based on the equal emphasis on environmental protection and economic development. Reasonable environmental regulation can not only promote the development of a green economy, but also be more conducive to the support of national policies, which has strong practical significance. To sum up, this paper makes policy analysis on three types of manufacturing industries respectively.

In order to comply with the requirements of the Central Economic Work Conference in 2020, for the high pollution industries in the manufacturing industry that is in urgent need to reduce the intensity of environmental regulation, industry should start within itself, optimize the industrial structure, keep accelerating economic development and strengthening environmental protection, and establish the development mode of the circular economy. It is suggested that the efficiency of resource utilization be improved, pressure on the ecological environment is reduced, scientifically formulate capacity replacement plans are scientifically formulated, and low-end excess capacity is transformed into high-end capacity that meets the market demand. We should reasonably and orderly reduce industries with high energy consumption, high pollution, low output and low efficiency, achieve the goal of resource-intensive development with low input, high output and low pollution so as to realize the unification of economic benefit, social benefit and environmental benefit of high energy consuming industries.

The light pollution industry is currently dominated by technology industries. We should deepen the supply-side reform and comprehensively improve quality and efficiency. The goal of “carbon peak” and “carbon neutrality” has set new goals and injected new impetus into China’s low-carbon development in the new stage. Under such a back-ground, we should promote the structural adjustment of light pollution industries by means of reform, take reducing carbon emissions as the main goal, reduce ineffective and low-end supply, expand effective and medium- and high-end supply, improve TFP, and realize the dynamic balance between supply and demand. Through environmental regulation, the proportion of low-tech and low value-added industries will be gradually reduced, so that those industries embodying new technology, new directions and new energy will gradually occupy an advantageous and dominant position, and promote the establishment of a development pattern of industry towards the medium-high end.

The current intensity of environmental regulation in moderate pollution industries is at a reasonable level. First, the growth rate of TFP has a strong correlation with that of GDP, and it is necessary to ensure the moderate TFP growth in the industry. Some industries with strong environmental regulations are allowed to improve their economic benefits by increasing energy consumption (reflected in carbon emissions). Second, for some industries with weak environmental regulation, while ensuring economic benefits, set strict carbon emission requirements. Reduce carbon emissions and strengthen environmental regulations by promoting pricing mechanisms for relatively low-carbon energy such as electricity, natural gas and pipeline gas. Setting the intensity of environmental regulation with carbon emissions as an important indicator in the manufacturing industry will re-shape the production mode of the manufacturing industry and have a broad and far-reaching impact on future economic and social development.

## Figures and Tables

**Table 1:** Interpretation of main data.

Model	Data	Variable	Source and Interpretation
SBM-MALMQUIST	Expected output	Desirable output	Using the total industrial output value of 27 manufacturing industries in 2009–2015. The basic data comes from *China Industrial Statistical Yearbook*, which have been converted into the constant price in 2000 according to the price index.
	Unexpected output	Undesired output	In order to measure the green economy efficiency of manufacturing industry more reasonably, this paper selects 27 items of manufacturing wastewater, solid waste and waste gas (carbon dioxide, sulfur dioxide) as the undesirable outputs. (In MAXDEA, data can not be identified as unexpected output, so the above three types of data are counted into the expected output in a negative way, equivalent to the unexpected output). The above data are obtained from *China Industrial Statistical Yearbook*.
SBM-MALMQUIST	Investment	Input	Previous scholars set the input variables as capital input and labor input but lack consideration for environmental resource consumption. Therefore, this paper puts the consumption of environmental resources in manufacturing industry into the category of efficiency measure, that is, adding the data of energy input.
	Capital stock	Capital stock	As an important variable in studying GTFP, there is no direct data. Estimate is required. As an important variable in the study of GTFP, there is no direct data, which requires estimate. This paper adopts the perpetual inventory method, taking 2008 as the base year of capital stock, referring to the calculation data of Brandt [37]. The depreciation rate is calculated according to the data of *China Industrial Statistical Yearbook* in 2009 to 2015. The constant price of current year investment is obtained using the difference between the original value of fixed assets to construct the sequence of investment amount, and converting it into the constant price of 2008 according to the price index of investment goods of the year as the investment amount of that year.
Regression model	Green total factor productivity	GTFP	According to the SBM-MALMQUIST model, GTFP of 27 manufacturing industries in 2010 to 2015 is obtained by MAXDEA.
	Direct carbon footprint	CF	According to the energy consumption data in the *China Energy Statistical Yearbook*, the main energy consumption of various industries in China is selected. Using a unified conversion standard, the energy consumption is converted into standard coal consumption, which is the direct carbon footprint discussed in this paper.
Regression model	Environmental regulation intensity	ER	The accurate measurement of environmental regulation intensity is the basis of empirical research on environmental regulation and GTFP. This paper establishes a measurement index system, and the environmental regulation intensity of each industry is obtained by weighted average of the data indicators such as the standard rate of wastewater discharge, the removal rate of waste gas and the comprehensive utilization rate of solid waste.
	Ratio of cost-profit	RCP	It reflects the ratio of cost input and profit of each industry, and the cost of each industry can be obtained in the statistical yearbook.
	Full-staff labor productivity	LP	The ratio of industrial added value to all employees in the corresponding industry reflects the average value created in each industry per capita every year. The data are all from *China Industrial Statistical Yearbook.*
	Energy productivity	EP	The ratio of industrial added value to the energy consumption of the corresponding industry. The data are all from *China Industrial Statistical Yearbook.*

**Table 2:** GTFP of various industries.

Industry/Year	2010	2011	2012	2013	2014	2015
Food processing industry	1.000	1.000	1.000	0.627	0.771	0.797
Food manufacturing industry	0.519	0.550	0.532	0.458	0.479	1.000
Beverage manufacturing industry	1.000	1.000	1.000	0.566	0.618	0.660
Tobacco processing industry	1.000	1.000	1.000	1.000	1.000	1.000
Textile industry	0.386	0.354	0.366	0.294	0.347	0.432
Manufacturing industry of clothing and other fiber products	0.304	0.309	0.179	0.121	0.199	0.186
Industry of leather fur down and their products	1.000	1.000	1.000	1.000	1.000	1.000
Wood processing and bamboo, rattan and palm grass products industry	0.158	0.146	0.170	0.096	0.298	0.373
Furniture manufacturing industry	0.246	1.000	0.162	0.089	0.075	0.085
Paper making and paper products industry	1.000	1.000	1.000	1.000	1.000	1.000
Reproduction of print recording media	0.065	0.079	0.076	0.058	0.097	0.146
Culture, educational and sports goods manufacturing industry	0.117	0.154	0.047	0.042	0.037	1.000
Petroleum processing and coking industry	1.000	1.000	1.000	1.000	1.000	1.000
Chemical raw materials and products manufacturing industry	0.625	1.000	1.000	0.514	0.703	1.000
Pharmaceutical manufacturing industry	0.450	0.439	0.453	0.321	0.377	0.387
Chemical fiber manufacturing industry	0.706	0.622	0.577	0.437	0.561	0.575
Rubber products industry	0.141	0.158	0.171	0.201	0.211	0.306
Non-metallic mineral products industry	1.000	0.172	0.214	0.243	0.460	1.000
Ferrous metal smelting and calendering industry	1.000	1.000	1.000	1.000	1.000	1.000
Nonferrous metal smelting and calendering industry	1.000	1.000	1.000	1.000	1.000	1.000
Metal products industry	0.191	0.238	0.242	0.269	0.240	0.330
General machinery manufacturing industry	0.145	0.095	0.080	0.064	0.086	0.085
Special equipment manufacturing industry	0.169	0.113	0.134	0.096	0.125	0.115
Transportation equipment manufacturing industry	0.289	0.138	0.148	0.133	0.161	0.181
Electrical machinery and equipment manufacturing industry	1.000	0.216	0.104	0.078	0.149	0.142
Electronic and communication equipment manufacturing industry	0.207	1.000	0.209	0.083	0.136	0.114
Instruments and meters, office machinery	0.205	0.118	0.068	0.040	0.093	0.070

**Table 3:** The hierarchical analysis system.

Target Level	Criterion Level	Scheme Level
Environmental regulation intensity	Wastewater	Discharge amount of wastewater
		Amount of wastewater that reaches the standard
	Solid waste	Discharge amount of solid waste
		Utilization of solid waste
	Waste gas	Amount of waste gas treatment equipment
		Amount of waste gas emission

**Table 4:** Calculation of environmental regulation intensity in different industries and years.

Industry/Year	2009	2010	2011	2012	2013	2014	2015
Food processing industry	0.423	0.319	0.423	0.422	0.479	0.497	0.154
Food manufacturing industry	0.147	0.413	0.396	0.261	0.280	0.347	0.205
Beverage manufacturing industry	0.389	0.384	0.792	0.503	0.530	0.650	0.140
Tobacco processing industry	0.151	0.053	0.128	0.059	0.047	0.066	0.054
Textile industry	1.389	1.472	1.002	1.072	1.179	1.295	0.256
Manufacturing industry of clothing and other fiber products	2.600	0.044	0.075	0.060	0.057	0.057	0.035
Industry of leather fur down and their products	0.366	0.476	0.245	0.296	0.269	0.241	0.195
Wood processing and bamboo, rattan and palm grass products industry	0.109	0.038	0.089	0.069	0.056	0.075	0.035
Furniture manufacturing industry	0.780	0.128	0.076	0.050	0.040	0.062	0.090
Paper making and paper products industry	7.456	8.700	20.040	10.614	8.741	8.733	2.695
Reproduction of print recording media	0.322	0.500	0.104	0.149	0.118	0.132	0.248
Culture, educational and sports goods manufacturing industry	0.085	0.090	0.211	0.334	0.073	0.151	0.039
Petroleum processing and coking industry	2.351	1.710	0.832	0.569	0.709	1.783	2.039
Chemical raw materials and products manufacturing industry	3.485	2.656	1.964	2.438	2.668	3.546	2.155
Pharmaceutical manufacturing industry	1.509	1.154	0.827	1.522	1.552	1.268	1.618
Chemical fiber manufacturing industry	3.830	0.894	3.257	0.888	1.523	1.558	6.378
Rubber products industry	0.213	0.154	0.636	0.694	0.656	1.001	1.066
Non-metallic mineral products industry	0.483	0.366	0.596	0.833	0.598	0.432	0.407
Ferrous metal smelting and calendering industry	0.536	0.832	0.659	0.693	0.721	1.095	1.199
Nonferrous metal smelting and calendering industry	0.198	0.131	1.749	0.267	0.138	0.325	0.331
Metal products industry	1.456	1.352	0.156	0.904	1.438	2.000	1.172
General machinery manufacturing industry	0.214	0.156	0.090	0.163	0.183	0.173	0.195
Special equipment manufacturing industry	0.112	0.104	0.051	0.114	0.104	0.148	0.129
Transportation equipment manufacturing industry	0.358	0.370	0.267	0.429	0.595	0.528	0.467
Electrical machinery and equipment manufacturing industry	0.175	0.181	0.176	0.258	0.339	0.337	0.229
Electronic and communication equipment manufacturing industry	0.524	0.461	0.588	1.015	0.397	0.103	0.031
Instruments and meters, office machinery	0.343	0.015	0.149	0.263	0.175	0.263	0.107

**Table 5:** Benchmark regression results.

	CF (14)	GTFP (15)	GTFP (16)	GTFP (17)	GTFP (18)	GTFP (19)
C.	6.323 *** (40.302)	0.460 *** (14.858)	0.009 (0.919)	0.162 (1.156)	0.189 (1.345)	0.144 (1.060)
ER.	0.302 *** (2.649)	0.050 *** (3.864)	0.031 *** (2.449)	0.031 *** (2.479)	0.029 ** (2.278)	0.019 * (1.628)
CF.			0.073 *** (5.329)	0.067 *** (4.651)	0.065 *** (4.577)	0.079 *** (5.544)
RCP.				−0.015 * (1.411)	−0.011 (−0.981)	−0.007 (−0.700)
LP.					−0.007 * (−1.762)	−0.006 (−1.569)
EP.						−0.020 *** (−3.617)

***, ** and * indicate significant at the level of 1%, 5% and 10% respectively. The numbers in parentheses on the first row of the table represent the corresponding equations.

**Table 6:** Descriptive statistics of the regression variables.

Industry Category	Variable	Number of Observations	Average	Standard Deviation	Minimum Value	Maximum Value
Light pollution industry	TFP	54	0.480	0.376	0.040	1.000
	ER	54	0.263	0.380	0.040	2.600
	CF	54	5.519	1.349	3.020	7.720
	RCP	54	5.885	7.506	1.010	33.830
	EP	54	2.813	1.159	1.280	5.91
	LP	54	10.709	7.775	4.770	34.73
Moderate pollution industry	TFP	60	0.362	0.348	0.040	1
	ER	60	0.446	0.337	0.020	1.470
	CF	60	6.062	1.914	3.290	10.900
	RCP	60	3.126	1.957	0.210	7.94
	EP	60	14.001	7.157	3.170	31.5
	LP	60	8.101	2.176	2.360	13.78
Heavy pollution industry	TFP	54	0.704	0.335	0.080	1
	ER	54	2.343	3.436	0.100	20.040
	CF	54	7.764	1.91	4.900	11.090
	RCP	54	1.337	1.641	0.110	5.96
	EP	54	3.057	0.544	2.200	4.33
	LP	54	6.079	2.762	0.810	13.15

**Table 7:** Regression results by classification.

	Light Pollution Industry			Moderate Pollution Industry			Heavy Pollution Industry	
	ER	TFP (1)	TFP (2)	ER	TFP (1)	TFP (2)	ER	TFP
C	5.351 (26.442)	−0.165 (−1.209)	−0.424 ** (−2.310)	4.927 (13.332)	−0.199 (−0.844)	−1.451 *** (−3.088)	7.6 (25.352)	−0.024 (−0.114)
CF	1.309 * (1.613)		0.090 ** (2.035)	0.388 *** (3.755)		0.144 *** (3.015)	1.471 (0.894)	0.055 ** (1.898)
ER		1.188 *** (4.062)	1.170 *** (4.091)		1.208 ** (2.461)	0.824 * (1.734)		0.046 *** (3.503)
ER^2^		−0.430 *** (−3.548)	−0.434 *** (−3.692)		−0.722 ** (−2.106)	−0.466 (−1.410)		−0.054 *** (−2.520)
RCP		0.014 (0.768)	0.025 (1.357)		0.044 * (1.838)	0.099 *** (3.434)		−0.001 (−0.051)
LP		0.090 (0.375)	−0.072 (−0.573)		−0.010 (−1.347)	−0.033 *** (−3.169)		0.113 (1.428)
EP		0.003 (0.247)	0.021 (1.358)		0.012 (0.423)	0.119 *** (2.714)		−0.096 *** (−4.038)
Inflection point		1.381	1.348		0.837	0.884		0.426

***, ** and * indicate significant at the level of 1%, 5% and 10% respectively.

## Data Availability

China Industrial Statistical Yearbook, China Energy Statistical Yearbook.
